# Use of JYNNEOS (Smallpox and Monkeypox Vaccine, Live, Nonreplicating) for Preexposure Vaccination of Persons at Risk for Occupational Exposure to Orthopoxviruses: Recommendations of the Advisory Committee on Immunization Practices — United States, 2022

**DOI:** 10.15585/mmwr.mm7122e1

**Published:** 2022-06-03

**Authors:** Agam K. Rao, Brett W. Petersen, Florence Whitehill, Jafar H. Razeq, Stuart N. Isaacs, Michael J. Merchlinsky, Doug Campos-Outcalt, Rebecca L. Morgan, Inger Damon, Pablo J. Sánchez, Beth P. Bell

**Affiliations:** ^1^Division of High Consequence Pathogens and Pathology, National Center for Emerging and Zoonotic Infectious Diseases, CDC; ^2^Epidemic Intelligence Service, CDC; ^3^Connecticut State Public Health Laboratory, Rocky Hill, Connecticut; ^4^Perelman School of Medicine, University of Pennsylvania, Philadelphia, Pennsylvania; ^5^Biomedical Advanced Research and Development Authority, U.S. Department of Health and Human Services, Washington, DC; ^6^University of Arizona College of Medicine, Phoenix, Arizona; ^7^Department of Health Research Methods, Evidence and Impact, McMaster University, Hamilton, Ontario, Canada; ^8^Nationwide Children’s Hospital, The Ohio State University College of Medicine, Columbus, Ohio; ^9^University of Washington, Seattle, Washington.

Certain laboratorians and health care personnel can be exposed to orthopoxviruses through occupational activities. Because orthopoxvirus infections resulting from occupational exposures can be serious, the Advisory Committee on Immunization Practices (ACIP) has continued to recommend preexposure vaccination for these persons since 1980 (*1*), when smallpox was eradicated (*2*). In 2015, ACIP made recommendations for the use of ACAM2000, the only orthopoxvirus vaccine available in the United States at that time (*3*). During 2020–2021, ACIP considered evidence for use of JYNNEOS, a replication-deficient *Vaccinia virus* vaccine, as an alternative to ACAM2000. In November 2021, ACIP unanimously voted in favor of JYNNEOS as an alternative to ACAM2000 for primary vaccination and booster doses. With these recommendations for use of JYNNEOS, two vaccines (ACAM2000 and JYNNEOS) are now available and recommended for preexposure prophylaxis against orthopoxvirus infection among persons at risk for such exposures.

Orthopoxviruses are large, double-stranded DNA viruses (Genus *Orthopoxvirus*, Family Poxviridae) that comprise multiple species, including *Variola virus*, *Vaccinia virus*, *Monkeypox virus*, *Cowpox virus*, and newly discovered species (e.g., *Akhmeta virus* and *Alaskapox virus*) (*4*). Infection with an orthopoxvirus or immunization with an orthopoxvirus vaccine lends immunologic cross-protection against other viruses in the genus (*3*). Until 1971, children in the United States received an orthopoxvirus vaccine (to prevent smallpox) as part of their routine childhood vaccines. However, with the World Health Organization (WHO) declaration of the eradication of smallpox (the infection caused by *Variola virus*) in 1980 (*2*), recommendations for routine vaccinations ended worldwide.

A small subset of persons in the United States continues to receive orthopoxvirus vaccination (*3*): persons at occupational risk for exposure to orthopoxvirus infections and certain U.S. military personnel. The first group (those with occupational risk for exposure) are within the purview of ACIP and the focus of this report. Regular booster doses are recommended for persons with ongoing occupational risk for exposure to orthopoxvirus infections. Designated public health and health care worker response teams approved by public health authorities should receive booster vaccination only at the time of an event, rather than at regular intervals.*

Poxviruses are increasingly being used in a wide range of biomedical research (*3*). *Vaccinia virus* is the most frequently studied poxvirus and serves as the prototype of the orthopoxvirus genus. This orthopoxvirus is used in basic virologic research, and because of its ability to serve as a vector for the expression of foreign genetic material, it is often used as an immunology tool and potential vaccine vector. *Vaccinia virus* is considered one of the less virulent orthopoxviruses, and possibly because of this perception, many laboratorians who work with this virus do not receive preexposure prophylaxis. CDC has received reports of occupational exposures to *Vaccinia virus* over the years and in some cases, morbidity has not been insignificant (*5*,*6*) In nearly all cases, infections with *Vaccinia virus* occurred in persons who were unvaccinated or previously vaccinated but not up to date with recommended booster doses.

In addition to less virulent viruses like *Vaccinia virus*, some researchers work with more virulent orthopoxviruses, including *Variola virus* (in some CDC laboratories) and *Monkeypox virus.* ACIP has historically recommended more frequent booster vaccination doses for persons working with more virulent orthopoxviruses than for those working with less virulent orthopoxviruses (*3*).

Replication-competent poxvirus strains can cause clinical infection in humans as well as produce infectious virus that can be transmitted to others (*3*). Replication-deficient poxvirus strains, including modified vaccinia Ankara (MVA), TROVAC, and ALVAC, do not produce infectious virus in humans, and therefore do not cause clinical infection; as such, replication-deficient poxvirus strains pose a substantially lower risk of adverse events compared with replication-competent strains. During 2015–2019, ACAM2000 was the only orthopoxvirus vaccine licensed by the Food and Drug Administration (FDA); ACIP recommendations for use of ACAM2000 in the United States were published in 2015 (*3*). ACAM2000 is a replication-competent *Vaccinia virus* vaccine derived from a plaque-purified clone of the same New York City Board of Health strain that was used to manufacture Dryvax vaccine, one of the vaccines used in the eradication of smallpox. Because ACAM2000 is replication-competent, there is a risk for serious adverse events (e.g., progressive vaccinia and eczema vaccinatum) with it; myopericarditis also occurs with ACAM2000 (estimated rate of 5.7 per 1,000 primary vaccinees based on clinical trial data), but the underlying mechanism is unknown (*7*,*8*).

In 2019, FDA licensed JYNNEOS, a replication-deficient MVA vaccine, for prevention of smallpox or monkeypox disease in adults aged ≥18 years determined to be at high risk for infection with these viruses. JYNNEOS is administered by subcutaneous injection as a 2-dose series delivered 28 days apart. There is no major cutaneous reaction, also known as a “take” (a vaccine site lesion often used as a marker of successful vaccination with replication-competent vaccines such as ACAM2000), following vaccination with JYNNEOS and consequently no risk for inadvertent inoculation or autoinoculation. The effectiveness of JYNNEOS was inferred from the immunogenicity of JYNNEOS in clinical studies and from efficacy data from animal challenge studies. Occurrences of serious adverse events are expected to be minimal because JYNNEOS is a replication-deficient virus vaccine. However, because the mechanism for myopericarditis following receipt of ACAM2000 is thought to be an immune-mediated phenomenon, it is not known whether the antigen or antigens that precipitate autoantibodies are present in JYNNEOS as well. ACIP began considering discussing the data for JYNNEOS in February 2020. This report describes the ACIP recommendations for the use of JYNNEOS for preexposure prophylaxis in persons at occupational risk for exposure to orthopoxviruses.

## Methods

During January 2020–November 2021, the ACIP Orthopoxvirus Work Group participated in monthly or bimonthly teleconferences to consider the evidence for five questions: 1) should JYNNEOS be recommended for research laboratory personnel, clinical laboratory personnel performing diagnostic testing for orthopoxviruses, and designated response team members at risk for occupational exposure to orthopoxviruses; 2) should JYNNEOS be recommended for health care personnel who administer ACAM2000 or care for patients infected with replication-competent orthopoxviruses; 3) should persons who are at ongoing risk for occupational exposure to more virulent orthopoxviruses such as *Variola virus* or *Monkeypox virus* receive a booster dose of JYNNEOS every 2 years after the primary JYNNEOS series; 4) should persons who are at ongoing risk for occupational exposure to less virulent replication-competent orthopoxviruses such as *Vaccinia virus* or *Cowpox virus* receive a booster dose of JYNNEOS at least every 10 years after the primary JYNNEOS series; and 5) should persons who are at ongoing risk for occupational exposure to orthopoxviruses and who received an ACAM2000 primary vaccination have the option to receive a booster dose of JYNNEOS as an alternative to a booster dose of ACAM2000. The Work Group comprised experts in diverse disciplines, including laboratory, public health, infection control, preparedness, and various clinical specialties (e.g., infectious disease, obstetrics, and occupational health). Federal partners represented on the Work Group included FDA, the National Institutes of Health, the U.S. Department of Defense, and the U.S. Department of Health and Human Services-Biomedical Advanced Research and Development Authority. CDC contributors also joined Work Group meetings with subject matter expertise in orthopoxviruses, regulatory affairs, laboratory diagnostic testing, vaccine adverse events, and drug services. Data collected, analyzed, and prepared by the Work Group were deliberated by ACIP during four public meetings.

Subject matter experts performed a systematic review and metaanalysis of the published literature on August 12, 2020, to inform the recommendations; the review was not limited by date or language. The Work Group used a modified Grading of Recommendations Assessment, Development and Evaluation (GRADE) approach to determine the certainty of evidence rated on a scale of 1 (high certainty) to 4 (very low certainty) for the following desirable and undesirable outcomes deemed critical for decision-making: prevention of disease, incidence of serious adverse events, and incidence of myopericarditis; prevention of disease was defined as prevention of an orthopoxvirus infection. Although no level of antibody protection for orthopoxviruses has been established, the detection of neutralizing antibodies after JYNNEOS is an indirect measure of protection (i.e., immunogenicity). Immunogenicity that peaks 2 weeks after completion of the 2-dose series (i.e., 6 weeks after the first vaccine in the 2-dose series) is called primary immunogenicity. Within the evidence to recommendations (EtR) framework, ACIP considered the importance of orthopoxvirus infection as a public health problem; the benefits and harms (including the graded evidence); the target populations’ values and preferences; and issues of resource use, acceptability to stakeholders, feasibility of implementation, and anticipated impact on health equity.

## Summary of Findings and Rationale for Recommendations

For the first and second questions, regarding recommendation for JYNNEOS as an alternative to ACAM2000 for primary vaccination, the systematic review identified three randomized controlled studies and 15 observational studies including a total of 5,775 subjects. After considering geometric mean titers and seroconversion data together, the Work Group had moderate (level 2) certainty that JYNNEOS provides a small increase in disease prevention compared with that provided by ACAM2000.^†^ The Work Group estimated with low (level 3) certainty that fewer serious adverse events occur following the JYNNEOS primary series compared with ACAM2000 primary vaccination, and that fewer events of myopericarditis occur after JYNNEOS primary series than after ACAM2000 primary vaccination. Based on the results from the GRADE assessment and EtR framework,^§^ ACIP unanimously voted in favor of the JYNNEOS vaccine as an alternative to ACAM2000 for primary vaccination.

To address the third and fourth questions, regarding booster doses, the systematic review identified one randomized controlled trial and 17 observational studies that included a total of 6,417 subjects. After considering geometric mean titer and seroconversion rate together, the Work Group estimated with very low (level 4) certainty that a small increase in disease prevention occurs after JYNNEOS booster versus the JYNNEOS primary series only.^¶^ The Work Group estimated with very low (level 4) certainty that fewer serious adverse events occur after a JYNNEOS booster administered following completion of the JYNNEOS primary series compared with the JYNNEOS primary series (i.e., no booster dose). No myopericarditis events were recorded in either the intervention or comparison; for this reason, the effect was not estimable and the Work Group had very low (level 4) certainty that myopericarditis does not occur after JYNNEOS boosters because of inadequate sample size to detect rare events. The ACIP unanimously voted in favor of the JYNNEOS booster vaccine after the 2-dose JYNNEOS primary series. ACIP recommended that the JYNNEOS booster dose be administered every 2 years to persons working with more virulent orthopoxviruses and every 10 years to persons working with less virulent orthopoxviruses.

For the fifth question, regarding providing the option of transitioning to JYNNEOS for a booster dose in persons who had received primary vaccination with ACAM2000, the systematic review identified one randomized controlled trial and five observational studies that included a total of 435 subjects. A total of 82% of subjects seroconverted when given JYNNEOS booster, with very low (level 4) certainty in that estimate. The Work Group estimated, with low (level 3) certainty, fewer serious adverse events occurred after the JYNNEOS booster than after the ACAM2000 booster in persons previously vaccinated with ACAM2000** and that fewer myopericarditis events occurred after a JYNNEOS booster than after an ACAM2000 booster in persons who received ACAM2000 as the primary vaccine (very low [level 3] certainty). Based on the results from the GRADE methodology and findings within the EtR framework,^††^ ACIP unanimously voted in favor of recommending JYNNEOS boosters as an alternative to ACAM2000 boosters in persons who received ACAM2000 as the primary vaccine.

## Recommendations

Research laboratory personnel,^§§^ clinical laboratory personnel performing diagnostic testing for orthopoxviruses,^¶¶^ designated response team members,*** and health care personnel who administer ACAM2000 (Smallpox [Vaccinia] Vaccine, Live)^†††^ or care for patients infected with orthopoxviruses^§§§^ are the persons to whom these recommendations apply (Table 1). For laboratory personnel and designated response team members, ACIP recommends use of JYNNEOS for primary vaccination as an alternative to ACAM2000. For health care personnel who administer ACAM2000 or care for patients infected with orthopoxviruses, ACIP recommends use of JYNNEOS (as an alternative to ACAM2000), based on shared clinical decision-making. In addition, persons who received the 2-dose JYNNEOS primary series and who are at ongoing risk^¶¶¶^ for occupational exposure to more virulent orthopoxvirus e.g., *Variola virus* and *Monkeypox virus*), should receive a booster dose of JYNNEOS every 2 years after the primary JYNNEOS series; persons who receive the 2-dose JYNNEOS primary series and who are at ongoing risk for occupational exposure to less virulent orthopoxviruses, (e.g., *Vaccinia virus* or *Cowpox virus*), should receive booster doses of JYNNEOS at least every 10 years after the primary JYNNEOS series. ACIP also recommends that persons who received an ACAM2000 primary vaccination and who are at ongoing risk for occupational exposure to orthopoxviruses may receive a booster dose of JYNNEOS as an alternative to a booster dose of ACAM2000.

**TABLE 1:** Recommendations for ACAM2000 and JYNNEOS vaccines for persons at occupational risk for exposure to orthopoxviruses — Advisory Committee of Immunization Practices, United States, 2022

Recommendations	Vaccine product	
	ACAM2000	JYNNEOS
Who should receive the vaccine?	Persons at risk for occupational exposure to orthopoxviruses*	
Who should be offered the vaccine?	Persons who administer ACAM2000 or care for patients with infection with replication-competent viruses	
Populations for whom booster vaccination is recommended at specific intervals	Persons who are at ongoing risk^†^ for occupational exposure to orthopoxviruses	
Booster frequency^§^		
Persons working with more virulent orthopoxviruses (e.g., *Variola virus* or *Monkeypox virus)*	Every 3 years	Every 2 years
Persons working with less virulent orthopoxviruses (e.g., *Vaccinia virus* or *Cowpox virus*)	At least every 10 years	

* Research laboratory personnel, clinical laboratory personnel performing diagnostic testing for orthopoxviruses, designated response team members, and health care personnel who administer ACAM2000 (Smallpox [Vaccinia] Vaccine, Live) or care for patients infected with orthopoxviruses.

^†^ Ongoing risk due to occupational work performed; response personnel are not considered at “sustained risk” for orthopoxvirus infections.

^§^ Booster doses are recommended for response personnel only once an event is identified.

## Clinical Guidance

### Considerations in Choosing JYNNEOS or ACAM2000 for Primary Vaccination

JYNNEOS involves a replication-deficient virus and has fewer contraindications, no risk for inadvertent inoculation and autoinoculation, and is associated with fewer serious adverse events compared with ACAM2000 (Table 2). In addition, most health care providers have experience with and are comfortable providing vaccines by subcutaneous administration, the route by which JYNNEOS is administered. ACAM2000, on the other hand, is administered percutaneously through a multiple puncture (scarification) technique, through 15 jabs with a stainless steel bifurcated needle that has been dipped into the reconstituted vaccine, a vaccination technique that is unique to orthopoxvirus vaccinations (*3*). JYNNEOS involves 2 vaccine doses 28 days apart and vaccine protection is not conferred until 2 weeks after receipt of the second dose; ACAM2000 involves 1 dose of vaccine and peak vaccine protection is conferred within 28 days.

**TABLE 2:** Distinctions between ACAM2000 and JYNNEOS that might facilitate decision-making among vaccinees at risk for orthopoxvirus infections — United States, 2022

Characteristic	Vaccine product	
	ACAM2000*	JYNNEOS
Vaccine virus	Replication-competent vaccinia virus	Replication-deficient modified vaccinia Ankara
“Take” following vaccination^†^	Yes	No
Risk for inadvertent inoculation and autoinoculation	Yes	No
Risk for serious adverse event	Yes	No significant events identified during clinical trials
Risk for cardiac adverse events	Myopericarditis in 5.7 per 1,000 primary vaccinees	Clinical trial data limited in evaluating this outcome; however, no significant events in data abstracted from single study arms^§^
Assessment of effectiveness	FDA assessed by comparing immunologic response and take rates to Dryvax*	FDA assessed by comparing immunologic response to ACAM2000 and animal studies
Administration	Percutaneously using a bifurcated needle by multiple puncture (scarification) technique,^¶^ single dose	Subcutaneously, 2 doses 28 days apart

**Abbreviation:** FDA = Food and Drug Administration.

*Both ACAM2000 and Dryvax are derived from the New York City Board of Health strain of vaccinia; ACAM2000 is a second generation smallpox vaccine derived from a clone of Dryvax, purified, and produced using modern cell culture technology.

^†^ A “take” is postvaccination lesion often used as a marker of successful vaccination after ACAM2000.

^§^ Because JYNNEOS is a replication-deficient virus vaccine, serious adverse events are believed to be fewer. However, the mechanism of myopericarditis in persons who receive ACAM2000 is poorly understood; for this reason, it is unknown whether persons who receive JYNNEOS might experience myopericarditis.

^¶^ https://www.fda.gov/media/75792/download

For those working with more virulent orthopoxviruses, the frequency of booster doses also differs: ACAM2000 boosters are recommended every 3 years, whereas JYNNEOS boosters are recommended every 2 years. After successful administration of vaccine, ACAM2000 produces a take containing infectious vaccinia virus capable of transmission through autoinoculation and inadvertent inoculation of close contacts of vaccinees; JYNNEOS does not produce a take. Some persons might prefer receiving ACAM2000 because the vaccine is a derivative of Dryvax, which was used successfully in eradicating smallpox, a clear demonstration of its effectiveness in preventing disease.

A robust antibody response following a single dose of JYNNEOS has been observed in clinical trials (*9*). In addition, limited data from animal model studies indicate that a single dose of JYNNEOS might provide protection for some persons against orthopoxvirus infection when administered before and closely after (1 day) viral challenge (*10*,*11*).

### Considerations for Transitioning from the Use of One Orthopoxvirus Vaccine to the Other for Booster Doses

Persons who previously received ACAM2000 should decide before their next booster dose whether to receive ACAM2000 or JYNNEOS. Persons who transition to receiving JYNNEOS boosters are expected to continue receiving JYNNEOS boosters and to not revert to ACAM2000; in addition, the frequency of booster doses should correspond to the vaccine used for boosters. For example, persons who previously received ACAM2000 every 3 years because of work with more virulent orthopoxviruses might decide to change to JYNNEOS when their next booster dose is due; in these cases, subsequent JYNNEOS booster doses should be administered every 2 years.

Fewer persons are expected to transition from JYNNEOS to ACAM2000; however, if those situations arise, they should be handled on a case-by-case basis. If this transition is approved by public health authorities, vaccinees should be advised that the expectation is that they will receive ACAM2000 for all future vaccine booster doses.

### Contraindications To and Precautions Associated with Vaccinations to Prevent Orthopoxvirus Infections

JYNNEOS is contraindicated in persons with a serious allergy to a vaccine component (Table 3). Primary vaccination with ACAM2000 is contraindicated in persons with the following conditions: serious allergy to a vaccine component, history of atopic dermatitis or other exfoliative skin condition,**** an immunocompromising condition (e.g., due to a disease or therapeutics),^††††^ pregnancy, breastfeeding, and known underlying heart disease (e.g., coronary artery disease or cardiomyopathy). ACAM2000 vaccination is also contraindicated if the vaccine recipient cannot sufficiently isolate from household contacts who have a history of atopic dermatitis or other active exfoliative skin condition, an immunocompromising condition, or who are pregnant or aged <1 year; household contacts include persons with prolonged intimate contact with the potential vaccine recipient and others who might have direct contact with the vaccination site or with potentially contaminated materials (e.g., clothing or vaccination site dressings). Availability of JYNNEOS provides opportunities for vaccinating persons in situations where ACAM2000 might be contraindicated.

**TABLE 3:** Contraindication to administration of ACAM2000 and JYNNEOS to recipients or their household contacts with certain conditions — United States, 2022

Clinical characteristic	Contraindication to receipt of ACAM2000			Contraindication to receipt of JYNNEOS
	Vaccine recipient with condition		Household contact with condition*	
	Primary vaccination	Revaccination		
History or presence of atopic dermatitis	Y	Y	Y	—
Other active exfoliative skin conditions^†^	Y	Y	Y	—
Immunosuppression^§^	Y	Y	Y	—
Pregnancy^¶^	Y	Y	Y	—
Age <1 year**	Y	Y	Y	—
Breastfeeding^††^	Y	Y	—	—
Serious vaccine component allergy	Y	Y	—	Y
Known underlying heart disease (e.g., coronary artery disease or cardiomyopathy)	Y	Y	—	—
≥3 known major cardiac risk factors^§§^	Y	—	—	—

**Abbreviation:** Y = yes.

* Household contacts include persons with prolonged intimate contact with the potential vaccinee (e.g., sexual contacts) and others who might have direct contact with the vaccination site or with potentially contaminated materials (e.g., dressings or clothing). JYNNEOS is a replication-deficient vaccine and therefore should not present a risk of transmission to household contacts.

^†^ Conditions include eczema, burns, impetigo, varicella-zoster, herpes, severe acne, severe diaper dermatitis with extensive areas of denuded skin, psoriasis, or Darier disease (keratosis follicularis). Studies evaluating JYNNEOS in persons with atopic dermatitis have demonstrated immunogenicity in eliciting a neutralizing antibody response and did not reveal any significant safety concerns.

^§^ Conditions include HIV; AIDS; leukemia; lymphoma; generalized malignancy; solid organ transplantation; therapy with alkylating agents, antimetabolites, radiation, tumor necrosis factor inhibitors, or high-dose corticosteroids; being a recipient of a hematopoietic stem cell transplant <24 months ago or ≥24 months ago but with graft-versus-host disease or disease relapse; or having autoimmune disease with immunodeficiency as a clinical component. Immunocompromised persons, including those receiving immunosuppressive therapy, may have a diminished immune response to JYNNEOS because of their immunocompromised status.

^¶^ Available human data on JYNNEOS administered to pregnant women are insufficient to determine vaccine-associated risks in pregnancy. However, animal models, including rats and rabbits, have shown no evidence of harm to a developing fetus.

** ACAM2000 is contraindicated in infants aged <1 year. Caution should be used when considering the administration of ACAM2000 or JYNNEOS to children and adolescents aged <18 years. JYNNEOS is not licensed for persons aged <18 years and has not been rigorously evaluated in this population.

^††^ The safety and efficacy of JYNNEOS has not been evaluated in breastfeeding women. It is not known whether JYNNEOS is excreted in human milk and data are not available to assess the impact of JYNNEOS on milk production or safety of JYNNEOS in breastfed infants. However, JYNNEOS vaccine is replication-deficient and therefore should not present a risk of transmission to breastfed infants. Caution should be used when considering the administration of JYNNEOS to breastfeeding women.

^§§^ Major cardiac risk factors include hypertension, diabetes, hypercholesterolemia, heart disease at age ≤50 years in a first-degree relative, and smoking. Clinical studies have not detected an increased risk of myopericarditis in recipients of JYNNEOS. Persons with underlying heart disease or ≥3 major cardiac risk factors should be counseled on the theoretical risk of myopericarditis given the uncertain etiology of myopericarditis associated with replication-competent smallpox vaccines.

Because of the documented risk for myocarditis after receipt of both ACAM2000 and mRNA COVID-19 vaccines (*12*) and the unknown risk for myocarditis after JYNNEOS, persons might consider waiting 4 weeks after orthopoxvirus vaccination (either JYNNEOS or ACAM2000) before receiving an mRNA COVID-19 vaccine, particularly adolescent or young adult males. However, if an orthopoxvirus vaccine is recommended for prophylaxis in the setting of an outbreak, administration of orthopoxvirus vaccine should not be delayed because of recent receipt of an mRNA COVID-19 vaccine. No minimum interval between mRNA COVID-19 vaccination and orthopoxvirus vaccination is necessary.

### Vaccinations Administered to Special Populations

**Persons with atopic dermatitis, eczema, or other exfoliative skin conditions**. Studies evaluating JYNNEOS in persons with atopic dermatitis have demonstrated immunogenicity in eliciting a neutralizing antibody response. No safety signals were revealed. However, persons with these conditions might be at increased risk for severe disease if an occupational infection occurs despite vaccination (*13*).

**Persons with immunocompromising conditions.** JYNNEOS is safe to administer to persons with immunocompromising conditions. However, such persons might be at increased risk for severe disease if an occupational infection occurs, despite vaccination. In addition, persons with immunocompromising conditions might be less likely to mount an effective response after any vaccination,^§§§§^ including after JYNNEOS; the risk/benefit ratio should be considered along with whether it is considered imperative to vaccinate an immunocompromised person.

**Pregnant women**. Available human data on JYNNEOS administered to pregnant women are insufficient to determine vaccine-associated risks in pregnancy. However, animal models, including rats and rabbits, have shown no evidence of harm to a developing fetus.

**Breastfeeding women**. The safety and efficacy of JYNNEOS has not been evaluated in breastfeeding women. It is not known whether JYNNEOS is excreted in human milk. Data are not available to assess the impact of JYNNEOS on milk production or the safety of JYNNEOS in breastfed infants. However, because JYNNEOS vaccine is replication-deficient, it likely does not present a risk of transmission to breastfed infants and can be administered to women who are breastfeeding if vaccination is critical.

**Children and adolescents aged <18 years.** Although occupational exposure to orthopoxviruses is unlikely in persons aged <18 years, it is important to note that JYNNEOS is not licensed for persons aged <18 years and has not been rigorously evaluated in this population. Public health authorities should be consulted if JYNNEOS is being considered for children and adolescents aged <18 years. Administration of ACAM2000 to infants aged <1 year is contraindicated. Caution should be used when considering the administration of ACAM2000 or JYNNEOS to children and adolescents aged <18 years.

**Persons with multiple cardiac risk factors**. Major cardiac risk factors include hypertension, diabetes, hypercholesterolemia, heart disease at age ≤50 years in a first-degree relative, and smoking and the presence of three or more of these factors are contraindications to primary vaccination with ACAM2000. Clinical studies have not detected an increased risk for myopericarditis in recipients of JYNNEOS. Persons with underlying heart disease or three or more major cardiac risk factors should be counseled about the theoretical risk for myopericarditis following vaccination with JYNNEOS given the uncertain etiology of myopericarditis associated with replication-competent smallpox vaccines such as ACAM2000.

### Reporting of Adverse Events

Adverse events following vaccination can be reported to the Vaccine Adverse Event Reporting System (VAERS). Reporting is encouraged for any clinically significant adverse event, even if it is uncertain whether the vaccine caused the event. Information on how to submit a report to VAERS is available at https://vaers.hhs.gov/index.html or by telephone at 1-800-822-7967.

### Peak Antibody Response, Confirming Effective Vaccination in Immunocompromised Persons, and Correlate of Protection After Vaccination with JYNNEOS

Peak antibody response is achieved 2 weeks after receipt of the second dose of the 2-dose JYNNEOS vaccination series (*9*). Evidence of a take is often used as a marker of successful vaccination after ACAM2000 (*3*). Because JYNNEOS is a replication-deficient vaccine, vaccination with JYNNEOS does not produce a take; however, clinical trials have demonstrated high rates of seroconversion after the 2-dose series. Therefore, effective vaccination can be assumed for immunocompetent persons. Routine antibody titer testing (to confirm successful vaccination) following vaccination with JYNNEOS is not recommended for immunocompetent persons. If the decision is made to vaccinate immunocompromised persons, titer testing by CDC might be considered on a case-by-case basis; clinicians considering vaccinating immunocompromised persons should consult public health authorities. Because a correlate of protection has not been established and there is no known antibody titer level that will ensure protection, titer results should be interpreted with caution in such cases to avoid providing a false sense of security. An immunocompetent person is considered fully immunized 2 weeks following administration of the second dose of the 2-dose JYNNEOS vaccination series, which is when clinical studies have demonstrated maximal antibody titers. Titer testing might also be considered on a case-by-case basis after vaccination of persons working with more virulent orthopoxviruses (e.g., *Variola virus* and *Monkeypox virus*).

### Minimizing Risk for Occupational Exposures

Many persons with contraindications to vaccination with ACAM2000 (e.g., atopic dermatitis, immunocompromising conditions, breastfeeding, or pregnancy) may receive vaccination with JYNNEOS. However, because the number of immunocompromised persons is increasing in the United States (*14*), and these persons might be less likely to mount an effective vaccine response, infections in vaccinated persons might occur. Outcomes after an infection in a vaccinated person could be particularly severe in these populations, particularly following exposure to more virulent orthopoxviruses (*3*); for this reason, vaccine recipients might consider avoiding high-risk exposures until after temporary conditions (e.g., pregnancy or transient therapy with immunocompromising therapeutics) are completed. If high-risk exposures cannot be avoided, persons who are pregnant, immunocompromised, or breastfeeding or who have atopic dermatitis may receive JYNNEOS in consultation with their health care provider and after careful consideration of the risks and benefits. Irrespective of vaccination status, all persons who work with orthopoxviruses should wear appropriate personal protective equipment.^¶¶¶¶^

## Future Research

Additional data on JYNNEOS vaccine are needed. Further studies are needed to determine the duration of protection after the 2-dose JYNNEOS vaccination series; recommendations regarding the frequency of booster doses can be modified accordingly. The effectiveness of a single dose JYNNEOS series should be evaluated if orthopoxvirus exposures occur before peak immunogenicity is achieved. Clinical trials evaluating the risk for myopericarditis and serious adverse events are needed to ensure that the risks are characterized and guidance about co-administration of JYNNEOS with mRNA COVID-19 vaccines can be elucidated. Establishing a correlate of protection after vaccination with JYNNEOS might facilitate confirmation of effective vaccination in certain populations and might also shed light on the effectiveness of a single dose of JYNNEOS vaccine. In addition, extensive studies to date have not identified the specific small mammal reservoir for some orthopoxviruses (e.g., *Monkeypox virus*); identifying the specific reservoir might facilitate the identification of high-risk activities for acquiring orthopoxvirus infections that are not already recognized.

**Summary**

**What is already known about this topic?**

In 2015, the Advisory Committee on Immunization Practices (ACIP) recommended preexposure prophylaxis with ACAM2000, a replication-competent live virus *Vaccinia virus* vaccine, for certain U.S. persons at risk for occupational exposure to orthopoxviruses.

**What is added by this report?**

In 2019, JYNNEOS, a replication-deficient live *Vaccinia virus* vaccine was licensed in the United States. On November 3, 2021, ACIP voted to recommend JYNNEOS preexposure prophylaxis as an alternative to ACAM2000 for certain persons at risk for exposure to orthopoxviruses.

**What are the implications for public health practice?**

A second vaccine is now available for persons for whom vaccination against orthopoxvirus infections is recommended. Potential vaccinees should weigh the risks and benefits of each vaccine when deciding which to receive.

## Footnotes

*
https://www.cdc.gov/smallpox/pdfs/revaccination-memo.pdf

†
https://www.cdc.gov/vaccines/acip/recs/grade/JYNNEOS-orthopoxvirus-primary-pq1-2.html

§ https://www.cdc.gov/vaccines/acip/recs/grade/JYNNEOS-orthopoxvirus-primary-pq1-etr.html; https://www.cdc.gov/vaccines/acip/recs/grade/JYNNEOS-orthopoxvirus-primary-hcp-etr.html

¶
https://www.cdc.gov/vaccines/acip/recs/grade/JYNNEOS-orthopoxvirus-booster.html

**
https://www.cdc.gov/vaccines/acip/recs/grade/JYNNEOS-orthopoxvirus-heterologous.html

††
https://www.cdc.gov/vaccines/acip/recs/grade/JYNNEOS-orthopoxvirus-heterologous-etr.htm l

§§ Research laboratory personnel are those who directly handle cultures or animals contaminated or infected with replication-competent vaccinia virus, recombinant vaccinia viruses derived from replication-competent vaccinia strains (i.e., those that are capable of causing clinical infection and producing infectious virus in humans), or other orthopoxviruses that infect humans (e.g., Monkeypox virus, Cowpox virus, and Variola virus).

¶¶ Clinical laboratory personnel who perform routine chemistry, hematology, and urinalysis testing, including for patients with suspected or confirmed orthopoxvirus infections, are not included in this recommendation because their risk for exposure is very low.

*** Public health authorities, at their own discretion, may approve a cohort of health care personnel, public health personnel, or both, to receive primary vaccination against orthopoxviruses for preparedness purposes (e.g., first responders who might participate in a smallpox or monkeypox outbreak).

†††
https://www.fda.gov/media/75792/download

§§§ For example, those caring for patients enrolled in clinical trials for replication-competent orthopoxvirus vaccines and those caring for persons with suspected or confirmed orthopoxvirus infections (e.g., clinicians and environmental services personnel).

¶¶¶ Continued risk refers to persistent risk due to occupational work performed. Designated public health and healthcare worker response teams approved by public health authorities are not at “continued risk” because they are vaccinated for the purposes of preparedness.

**** Examples include eczema, burns, impetigo, varicella-zoster, herpes, severe acne, severe diaper dermatitis with extensive areas of denuded skin, psoriasis, or Darier disease (keratosis follicularis).

†††† Conditions include HIV; AIDS; leukemia; lymphoma; generalized malignancy; solid organ transplantation; therapy with alkylating agents, antimetabolities, radiation, tumor necrosis factor inhibitors, or high-dose corticosteroids; being a recipient of a hematopoietic stem cell transplant <24 months ago or ≥24 months ago but with graft-versus-host disease or disease relapse; or having autoimmune disease with immunodeficiency as a clinical component.

§§§§
https://www.cdc.gov/vaccines/hcp/acip-recs/general-recs/immunocompetence.html

¶¶¶¶
https://www.cdc.gov/labs/BMBL.html
