# *Erratum:* Vol. 71, No. 22

**DOI:** 10.15585/mmwr.mm7127a5

**Published:** 2022-07-08

In the report, “Use of JYNNEOS (Smallpox and Monkeypox Vaccine, Live, Nonreplicating) for Preexposure Vaccination of Persons at Risk for Occupational Exposure to Orthopoxviruses: Recommendations of the Advisory Committee on Immunization Practices — United States, 2022,” on page 738, in Table 1, the first footnote should have read, “* Research laboratory personnel working with orthopoxviruses, clinical laboratory personnel performing diagnostic testing for orthopoxviruses, and orthopoxvirus and health care worker response teams designated by appropriate public health and antiterror authorities.”

